# Evaluating Treatment Outcomes for Pelvic Insufficiency Fractures: A Systematic Review

**DOI:** 10.3390/jcm13113176

**Published:** 2024-05-29

**Authors:** Giulia Maria Sassara, Amarildo Smakaj, Domenico De Mauro, Roberta Righini, Adele Arnone, Giuseppe Rovere, Omar El Ezzo, Pasquale Farsetti, Umberto Tarantino, Francesco Liuzza

**Affiliations:** 1 Department of Aging, Neurological, Orthopaedic and Head-Neck Sciences, Fondazione Policlinico Universitario Agostino Gemelli IRCCS, 00136 Rome, Italy; giuliamariasassara@gmail.com (G.M.S.); demaurodomenico@gmail.com (D.D.M.); adelearnone@gmail.com (A.A.); elezzo_omar@hotmail.it (O.E.E.); liuzzafrancesco@gmail.com (F.L.); 2 Department of Geriatrics and Orthopaedic Sciences, Università Cattolica del Sacro Cuore, 00168 Rome, Italy; rovere292@hotmail.com; 3 Department of Biomedicine and Prevention, “Tor Vergata” University of Rome, 00133 Rome, Italy; 4 Department of Orthopaedics and Traumatology, “Policlinico Tor Vergata” Foundation, 00133 Rome, Italy; pasquale.farsetti@ptvonline.it (P.F.); umberto.tarantino@ptvonline.it (U.T.); 5 Department of Public Health, Orthopedic Unit, Federico II University, 80131 Naples, Italy; 6 Department of Clinical Sciences and Translational Medicine, “Tor Vergata” University of Rome, 00133 Rome, Italy

**Keywords:** pelvic fracture, sacral fractures, insufficiency fracture, fragility fractures

## Abstract

**Background**: Pelvic insufficiency fractures (PIF) are typical in geriatric populations with reduced bone quality, most commonly in elderly postmenopausal women. These fractures are usually caused by low-energy forces over the bones during ordinary life and cause disabling pain. Treatment options range from conservative to operative. The aim of this study is to assess the outcomes of treatments for pelvic insufficiency fractures, determining optimal approaches between surgical intervention and conservative management. **Methods**: This literature review systematically examines articles focusing on patients with PIF, following the Preferred Reporting Items for Systematic Reviews and Meta-Analyses guidelines, and using PubMed, Medline, and the Cochrane Library database. We took into account only full-text articles in indexed journals with available English abstracts, considering data about patient demographics, surgery, and outcomes. **Results:** After screening 128 articles, this study reviewed 20 manuscripts involving 1499 patients, mostly elderly females and focusing on sacrum fractures. Common treatments included conservative methods and sacroplasty, with a few complications reported. Osteoporosis was the prevalent comorbidity, and the survival rate post-treatment was high at 92.3%. Mobility outcomes varied, with some patients experiencing significant autonomy loss. The average follow-up period was over 17 months. **Conclusions**: This study found a cautious approach to surgery (timing of three weeks), which is reserved only for specific patterns, and it leads to increased autonomy and a lower risk of mortality. Due to the lack of pre- and postoperative scores as well as conflicting results, it is imperative to undertake further studies and research to be able to compare the alternative treatments efficiently.

## 1. Introduction

Pelvic insufficiency fractures, usually caused by low-energy trauma during routine daily activities, represent a significant clinical challenge in the context of musculoskeletal health. They result from repetitive stresses acting upon bones with diminished structural integrity. These occurrences are particularly prevalent among elderly postmenopausal women [1] and are related to the natural decline in bone density associated with aging. Osteoporotic fractures, a subset of pelvic insufficiency fractures, tend to localize predominantly in specific anatomical regions, including the femoral neck, proximal humerus, wrist, and pelvic ring [2]. The aging demographic landscape, marked by an upward trend in average population age, exacerbates the prevalence and impact of these fractures, posing a growing burden on healthcare systems worldwide [3].

To better understand and manage pelvic insufficiency fractures, various classification systems have been developed over the years. Notable among these are Rommens’, Tile’s, and Young and Burgess classifications [1], each offering unique insights into fracture patterns and severity. These classification schemes serve as valuable tools for clinicians in diagnosing and stratifying fractures, thereby guiding treatment decisions and prognostic assessments.

The first challenge when treating PIFs is the different and often subtle clinical presentation, especially in those involving the sacrum that could end up being undiagnosed or misdiagnosed.

In the realm of treatment modalities for pelvic insufficiency fractures, a nuanced approach is warranted, taking into account fracture characteristics, patient comorbidities, and overall clinical context. Conservative management—characterized by enforced rest and pharmacological pain management [4]—remains a cornerstone in the management of non-displaced, inherently stable fractures, particularly in patients with multiple comorbidities or contraindications to surgical intervention. While conservative approaches may lead to shorter hospital stays, they are not without drawbacks including elevated rates of complications, mortality, persistent pain, and reduced patient satisfaction [5].

In cases where conservative measures prove insufficient or impractical, surgical intervention becomes necessary. Percutaneous stabilization techniques, such as the deployment of iliosacral or pelvic screws, have emerged as effective strategies for achieving fracture stability while minimizing surgical morbidity. These minimally invasive procedures are often complemented by adjunctive measures, including sacroplasty or balloon kyphoplasty [6], aimed at enhancing biomechanical support and facilitating fracture healing [7].

Sacroplasty, in particular, has garnered attention for its potential to mitigate complications associated with non-operative management. This percutaneous procedure offers several advantages, including shorter surgical duration, reduced blood loss, significant pain relief, and the potential for enhanced patient satisfaction [8]. However, concerns regarding cement leakage and nerve root injury underscore the importance of meticulous technique and patient selection in ensuring favorable outcomes.

Similarly, percutaneous screw fixation represents a viable option for patients with intolerable pain resulting from non-displaced fractures. While this approach allows for immediate mobilization and reduced hospitalization duration, it is not without risks including implant loosening and the need for re-operation [9].

The management of U-shaped insufficiency fractures presents unique challenges, often necessitating a multidisciplinary approach tailored to individual patient needs.

Combinations of sacroplasty and screw fixation may offer enhanced fracture stability and control over rotational forces, particularly in cases where traditional approaches fall short [10].

In instances of unstable or displaced fractures, conventional open reduction and internal fixation remain the gold standard [1] for achieving anatomical alignment and restoring pelvic integrity. While this approach offers advantages in terms of postoperative pain management and early mobilization [11], it is associated with prolonged hospitalization durations [12], underscoring the need for comprehensive preoperative assessment and patient counseling.

In summary, the management of pelvic insufficiency fractures requires a multifaceted approach, integrating conservative and surgical modalities tailored to individual patient characteristics and fracture patterns. Through an extensive review of existing literature, this study seeks to elucidate the criteria guiding the selection between surgical intervention and conservative treatments for pelvic insufficiency fractures. We aim to identify and analyze specific clinical scenarios where surgical interventions yield better outcomes than conservative treatments, ultimately leading to improved patient care and results.

## 2. Materials and Methods

**Figure 1:**
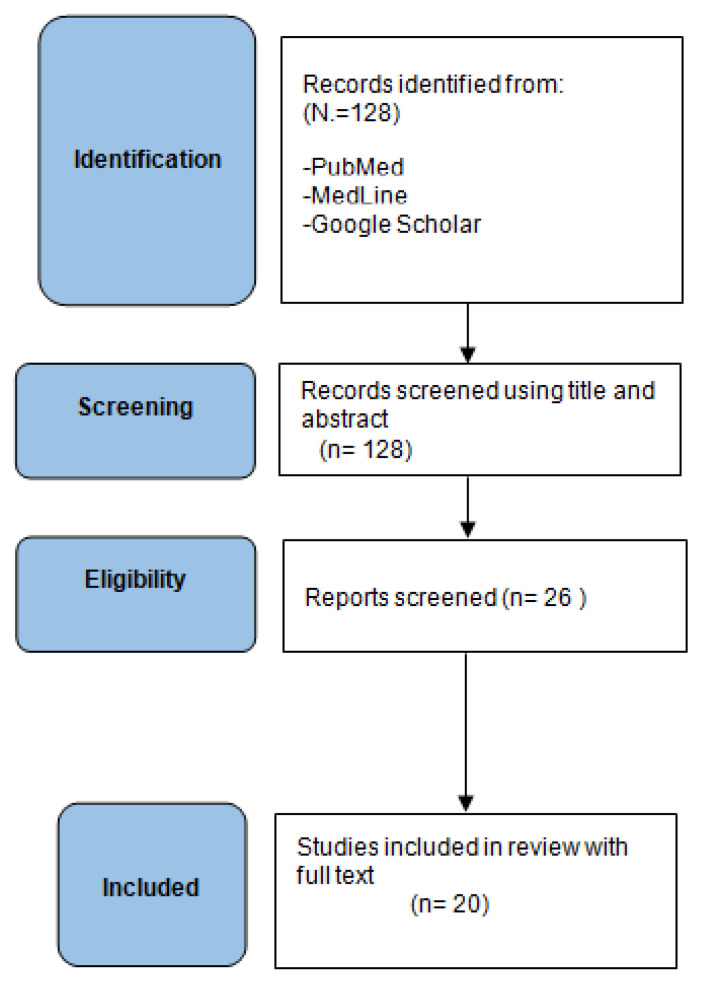
The PRISMA flowchart.

The present investigation represents a systematic literature review reported according to the Preferred Reporting Items for Systematic Reviews and Meta-Analyses (PRISMA) [13] guidelines and using PubMed, Medline and the Cochrane Library database. The digital search was conducted using specific keywords, their corresponding MeSH terms, and the logical operators “AND” and “OR”: (insufficiency sacral fractures OR sacral insufficiency fractures) AND (treatment OR management) AND (conservative OR non-operative) AND (operative OR surgery). The bibliography of the selected studies was meticulously searched by hand to identify additional studies that were not captured during the initial electronic search process. This comprehensive approach ensured that no potentially relevant study was overlooked. Importantly, there were no restrictions placed on the date of publication, allowing for a broad and inclusive collection of data. Moreover, at no point in the selection process were the journal titles, authors’ names, or names of supporting institutions concealed, ensuring transparency and allowing for a thorough assessment of all available literature. The PRISMA was followed as reported in Figure 1. This systematic review was not registered with any registry prior to its commencement. In this review, we considered the studies published as full-text articles in indexed journals and that investigated the treatment of pelvic insufficiency fractures, especially when there is a surgical treatment indication. Only articles written in English with available abstracts were included. No publication date limits were set. Surgical technique reports, expert opinions, letters to the editor, studies on animals, unpublished reports, cadaver or in vitro investigations, review of the literature, abstracts from scientific meetings, book chapters, and case reports were excluded from the present review.

Two independent reviewers (G.M.S. and A.S.) collected the data from the included studies. Any discordances were solved by consensus with a third author (F.L.). All abstracts were reviewed to determine adherence to inclusion and exclusion criteria of our study. If no abstract was published or if the abstract did not have sufficient information to determine eligibility, the full-length manuscript was reviewed. Articles with questionable data were discussed with the senior author. For each study included in the present analysis, the following data were extracted: main author, year of publication, article type, number of patients included, the sex of the enrolled patients, age, involvement of the sacrus, type of fracture, comorbidities, traumatic mechanism, type of surgery, time to first surgery, type of surgical fixation, complications, osteoporosis therapy, outcomes, and time of follow up. Given the heterogeneity of the studies examined, the statistical approach was limited to a descriptive analysis focusing on the collection and presentation of data. This decision was driven by the considerable variability in methodologies, populations, and outcomes across the studies, which inherently complicates the application of standard inferential techniques aimed at drawing general conclusions from data.

## 3. Results

The searches resulted in 128 articles. Following the PRISMA flow chart (Figure 1), 20 articles were considered relevant to the general topic area and were finally included in the review [2,5,9,10,14,15,16,17,18,19,20,21,22,23,24,25,26,27,28,29].

The studies included 1499 patients overall. About 88.6% of patients were female, and 16.5% of patients were male. Only two studies did not report the sex of the patients. The average age was about 73 years. The youngest patient was 18 years old, while the oldest was 99 years old. In all of the 20 studies, there was a sacrum involvement. The most common comorbidity was osteoporosis (reported by 11 authors), while malignancy (metastatic disease, endometrial carcinoma, advanced prostate carcinoma, and a pelvic radiation history) was reported only in three studies.

Only nine authors reported the traumatic mechanism causing the fracture, and low-energy trauma was the most common. Meanwhile, about 3.85% of the fractures were not related to trauma. Six studies reported conservative treatment options (25.35%). Sacroplasty was the most common surgical technique; only three authors described a technique under CT guidance. The median time to first surgery was three weeks. Only Eckardt et al. [9] reported a second surgery (0.6%). Except for one author [16], none reported preoperative or postoperative scores such as the Majeed score and the NRS. After surgery, in total, there were four types of cases of surgical technique complications: two cement leakages without symptoms (2.9%) in 73 cases, one cement leakage-related neurological complication (10%) in 10 cases, and two sacroiliac screws malposition (2.7%) in 73 cases. 

Nine authors reported on whether patients took osteoporosis therapy, but only three studies specified how many patients undertook it. The global survival rate was 92.3%, upon considering the occurrence of undesirable events after fractures such as stroke, heart failure, cancer, pneumonia, and infections. The preserved mobility of patients with conservative treatments was shown only by two authors: Maier et al. [2] reported that when the autonomous state before and after pelvic fractures was compared, a high loss of autonomy was observed; Na WC et al. noted that after the confirmation of bone union, full weight bearing was started [15]. The medium time of follow up was 17.43 months. Table 1 presents a summary of the key data analyzed.

## 4. Discussion

The review of 20 studies involving 1.499 patients provides treatment options and outcomes associated with pelvic insufficiency fractures [2,5,9,10,14,15,16,17,18,19,20,21,22,23,24,25,26,27,28,29]. The involvement of the sacrum in all 20 studies underscores the relevance of this region in PIFs [10,14,16]. The demographic data highlights a predominance of female patients (88.6%), emphasizing the higher susceptibility of women to PIFs. In fact, postmenopausal women are at a higher risk for primary osteoporosis, primarily because this condition is strongly associated with a deficiency in estrogen [30]. During the transition into menopause, a decrease in estrogen levels causes bones to weaken faster than they can be rebuilt, leading to the development of osteoporosis [31]. The prevalence of osteoporosis as the most common comorbidity confirms that PIFs often occur in individuals with compromised bone density [2,5,20,23]. Therefore, anti-osteoporotic therapy is of critical importance in preventing fractures and re-fractures. By strengthening bone mass and improving bone quality, appropriate drug treatments play a pivotal role in reducing the risk of fractures [32]. On the other hand, sarcopenia, which is characterized by being at the cut-off values for low muscle mass, strength, and/or functional capacity and associated with a range of metabolic conditions, shares common risk factors with osteoporosis, and are strongly associated with frailty, falls, fractures, hospitalizations, and mortality, as well as causing an upsurge in healthcare expenditures [33]. 

Tumor-related fractures were less frequent but represented an important sub-group among the diverse causes contributing to PIFs [20,22]. Trauma, particularly low-energy trauma, was identified as the leading cause in the studies reporting the mechanism, emphasizing the importance of recognizing PIFs in the absence of a clear traumatic background [15,26]. Older age, polypharmacy, malnutrition, frailty, smoking, and alcohol consumption significantly increased the risk of falls; these factors also reflect declines in physical condition [34]. Moreover, chronic illnesses are very common in older adults, and cardiac disease, hypertension, diabetes, stroke, and Parkinson’s disease are associated with falls. Older adults residing in urban areas had a higher risk of falling than those residing in rural areas [35]. Nevertheless, high-energy traumatic mechanisms leading to pelvic and acetabular fractures often involve numerous comorbidities and complications [36,37,38]. The treatment options revealed a preference for sacroplasty, with CT-guided techniques gaining space and attention [5,17,22,26,27,39]. In fact, minimally invasive percutaneous techniques allow for the fast and precise placement of the needle in and along the sacral wings, thus preventing the use of multiple needles to reach fracture sites [40]. Overall, sacroplasty has been shown to be an effective procedure in terms of pain relief. Early return to function and substantial pain relief distinguished this treatment modality from traditional methods of conservative management with physical therapy and oral analgesics. [41]. Staying on the subject of surgical treatment, Arduini et al. in 2015 showed encouraging results and demonstrated that minimal or less invasive osteosynthesis techniques could lead to good outcomes in these patients [42]. Conservative management was employed in a quarter of the cases, offering a non-invasive approach for selected patients [14,29]. This treatment option has some advantages in terms of reduced blood loss, infections, and hospitalization [43,44]. The timing of surgery, with a median of three weeks, suggests a careful approach to intervention [23,24]. However, the limited reporting of pre- and postoperative scores, except for a few studies, highlights the need for standardized outcome assessments in future research to facilitate significant comparisons. An interesting statement was made by Andresen et al. [5], who identified patients with a low level of pain as ideal candidates for conservative therapy. Adverse outcomes and the risk of death commonly rise when persistent immobilizing pain is present. According to them, surgical treatment can be recommended after considering fracture patterns: non-dislocated fractures are painful for patients, and they can receive benefits from sacroplasty; patients with unstable and displaced fractures can undergo osteosynthesis, leading to enhanced autonomy and diminished mortality. Complication rates, although relatively low in number, were recorded, especially those involving cement leakage [26,27]. Bastian et al. showed that leakage into the fracture gap is at high risk of affecting the 5th lumbar nerve root due to the special course of its ventral branch over the sacral promontory. Therefore, the risks of cement leakage with neurological impairment should be explained to patients [45]. This highlights the importance of precise technique and postoperative checks to decrease potential adverse events. The lack of consistent information on pre- and postoperative scores, as well as the lack of information on osteoporosis therapy, highlight the need for further and more complete studies to enhance the quality and comparability of evidence in this field. The overall survival rate of 92.3%, considering post-fracture events such as stroke, heart failure, cancer, pneumonia, and infections, underscores the impact of PIFs on patients’ health [6,17,24]. The recovery of mobility after conservative treatment has been analyzed by only a few authors [2,15], and conflicting results highlight the need for further investigation into the outcomes associated with different treatments. Sacral insufficiency fractures are certainly the most clinically relevant insufficiency fractures, but they are not the only ones. Pubic ramus fractures are generally considered stable insufficiency fractures and result from low-energy trauma and show a good prognosis [46]. However, several studies have described a connection between pubic ramus fractures and concurrent posterior pelvic ring injuries [47,48]. Elderly patients with pubic ramus fractures and concomitant posterior pelvic ring fractures have different characteristics than those with isolated ramus fractures [49]. When pubic ramus fractures are highly displaced, located medially to the obturator foramen, and involve complete anterior pelvic ring disruption, it is important to avoid misdiagnosing them as posterior insufficiency fractures [49].

The review’s strength is represented by its comprehensive overview of the current literature about a relevant topic. However, there are some limitations, such as the following: (i) the heterogeneity in the data reported, and (ii) the lack of standardized outcome measures, thus leaving space for improvement in future research. This review provides a valuable contribution for understanding PIFs, but further studies and long-term follow-up are mandatory to improve our knowledge and increase patient treatment choices.

## 5. Conclusions

PIFs often occur in elderly women with compromised bone density (with osteoporosis as the leading comorbidity). The sacrum has been revealed as the most relevant area involved in PIFs. Low-energy trauma is the principal mechanism in this type of fracture, and therefore, it is mandatory to investigate it in elderly patients with uncertain traumatic backgrounds. On the basis of the published literature we considered, this study demonstrated a cautious approach to surgery (timing of three weeks), which is reserved only for specific patterns, and it leads to increased autonomy and a lower risk of mortality; amongst the treatment options, there is a preference for sacroplasty rather than osteosynthesis. On the other hand, conservative treatment is reserved for patients with low levels of pain, even though this management often leads to a high risk of death as a result of immobilization. 

In conclusion, despite the extensive background in this review that provides a remarkable understanding of PIFs, further studies and investigations are required in order to discern the most appropriate treatment for patients.

## Figures and Tables

**Table 1:** Main demographical and clinical data from the included studies.

Article	Year	LoE	No. of Patients	M/F	Age	Comorbidity	Mechanism of Trauma	Surgical Treatment
Maier et al. [2]	2016	IV	93	40/53	70	Osteoporosis Hypertension Diabetes	-	N
Schramm et al. [14]	2023	III	46	4/42	83	-	-	N
Na et al. [15]	2017	III	15	1/14	76.5	Osteoporosis	Absence of trauma (n = 7) Minor trauma (n = 8)	3
Jackle et al.	2021	IV	72.70	32/51	73	-	Falling from a low high (n = 23), Falling in a domestic environment (n = 30), No trauma (n = 11), Unknown (n = 19)	Y
Pulley et al. [10]	2018	IV	18	-	75	-	Low-energy mechanisms	Y
Wahnert et al.	2013	IV	12	0/12	-	-	-	Y
Schmerwitz et al. [18]	2021	III	53	5/48	79.1		Low-energy trauma	Y
Eckardt et al. [9]	2017	III	50	7/43	79		Low-energy trauma	Y
Urzua et al. [19]	2011	III	42	10/32	40		Low-energy trauma (n = 32)	N
Kortman et al. [20]	2013	III	243	28/176	77.2	Osteoporosis (204), 39 infiltrative lesions	-	Y
Andresen et al. [5]	2022	III	292	16/276	80	Osteoporosis		-
Sandmann et al. [21]	2018	IV	8	0/8	83.1	Osteoporosis	-	Y
Heron et al. [22]	2007	IV	3	1/2	80	Osteoporosis, Metastatic disease, Endometrial carcinoma Advanced prostate carcinoma	-	Y
Andresen et al. [23]	2015	IV	20	2/18	80.4	Osteoporosis	-	Y
Andresen et al. [24]	2017	IV	23	3/20	81.3	Vitamin D deficiency	-	Y
Vanderschot et al. [25]	2009	IV	19	4/15	71.7	Osteoporosis Radiotherapy Rheumatoid arthritis	Minor trauma (n = 4)	Y
Heo et al. [26]	2017	III	68	4/64	76.8	Osteoporosis Radiotherapy Steroids therapy	Minor trauma (n = 32)	Y
Schwetje et al. [27]	2020	IV	10	3/7	78.4	Osteoporosis	-	Y
Park et al. [29]	2017	III	325	275/50	69.4	Malignancy, Pelvic radiation history, Hypertension, Diabetic mellitus, Ischemic heart disease, Stroke, Rheumatologic disease, Chronic kidney disease, Chronic liver disease, Endocrine disorder, Tuberculosis history	-	Y
Yoo et al. [28]	2017	III	41	5/36	74	-	-	N

## Data Availability

Dare available from the corresponding author on reasonable request.
